# Graphene Oxide Nanoribbons in Chitosan for Simultaneous Electrochemical Detection of Guanine, Adenine, Thymine and Cytosine

**DOI:** 10.3390/bios10040030

**Published:** 2020-03-27

**Authors:** Jiayun Zhou, Shaopei Li, Meissam Noroozifar, Kagan Kerman

**Affiliations:** Department of Physical and Environmental Sciences, University of Toronto Scarborough, 1265 Military Trail, Toronto, ON M1C 1A4, Canada; jiayun.zhou@mail.utoronto.ca (J.Z.); shaopei.li@mail.utoronto.ca (S.L.); m.noroozifar@utoronto.ca (M.N.)

**Keywords:** graphene oxide nanoribbons, nanocomposite, chitosan, DNA, nucleobases, voltammetry

## Abstract

Herein, graphene oxide nanoribbons (GONRs) were obtained from the oxidative unzipping of multi-walled carbon nanotubes. Covalent coupling reaction of 1-ethyl-3-(3-dimethylaminopropyl) carbodiimide hydrochloride (EDC) and N-hydroxy succinimide (NHS) with amine functional groups (-NH_2_) of the chitosan natural polymer (CH) was used for entrapping GONRs on the activated glassy carbon electrode (GCE/GONRs-CH). The nanocomposite was characterized by high-resolution transmission electron microscopy (HRTEM), and field-emission scanning electron microscopy (FESEM). In addition, the modification steps were monitored using FTIR. The nanocomposite-modified electrode was used for the simultaneous electrochemical determination of four DNA bases; guanine (G), adenine (A), thymine (T) and cytosine (C). The nanocomposite-modified GCE displayed a strong, stable and continuous four oxidation peaks during electrochemistry detection at potentials 0.63, 0.89, 1.13 and 1.27 V for G, A, T and C, respectively. The calibration curves were linear up to 256, 172, 855 and 342 μM with detection limits of 0.002, 0.023, 1.330 and 0.641 μM for G, A, T and C, respectively. The analytical performance of the GCE/GONRs-CH has been used for the determination of G, A, T and C in real samples and obtained a recovery percentage from 91.1%–104.7%. Our preliminary results demonstrated that GCE/GONRs-CH provided a promising platform to detect all four DNA bases for future studies on DNA damage and mutations.

## 1. Introduction

Deoxyribonucleic acid (DNA) bases are the fundamental building blocks of all life. There are four naturally found bases, with two subgroups. Guanine (G) and adenine (A) are in the group of purine bases in which the structure contains an aromatic pyrimidine ring fusing with an imidazole ring [1]. The second subgroup is the pyrimidines which are thymine (T) and cytosine (C), which only contain an aromatic pyrimidine ring within its structure [2]. By varying the sequence of these four bases, proteomic, metabolomic and physiological information can be stored and recalled at any time. In the human genomic sequence, if a mutation or damage occurs within the sequence it can lead to detrimental diseases such as cystic fibrosis, sickle cell anemia and Alzheimer’s disease [3,4,5]. Furthermore, many discovered disease pathogenesis pathways have a high correlation to mutations that occur within the genetic makeup. Thus, it is of a great interest to develop a biosensing platform that allows the analysis of the DNA bases. 

Various carbon-based surfaces such as graphite paste, glassy carbon and mesoporous carbon have been employed as the detection platform [6,7,8]. Brett et al. [9] performed the detection of all four DNA bases using a non-modified glassy carbon electrode (GCE). Other researchers achieved the attachment of two-dimensional nanomaterials such as MnO_2_ nanoparticles on GCE surfaces using direct electrodeposition to detect DNA bases [9]. However, to the best of our knowledge, a nanocomposite of chitosan (CH) and graphene oxide nanoribbons (GONRs) have yet to be explored for the simultaneous electrochemical determination of all four DNA bases. 

Carbon nanotubes (CNTs) have been found to be stable under ambient conditions and exhibit a strong ambipolar electric field which render them ideal for sensor development using electrochemistry [10]. Due to their high surface-to-volume ratio, they can drastically enhance the electron transport rate, which in turn enhances the overall conductivity. In addition, they are biocompatible and do not present a background signal when incorporated into electrochemical sensors [11]. Recently, GONRs have been synthesized by the unzipping of multi-walled carbon nanotubes (MWCNTs) combining the electrocatalytic properties of graphene oxide with the high surface-to-volume ratio of CNTs [12]. Unfortunately, this nanomaterial poses a challenge when in an attempt to incorporate it into the biosensor surface. One of the drawbacks is that traditional modification often employs simple drop-casting the materials onto the electrode. Though this approach is accessible and simple, simply drop-casting GONRs resulted in an unstable and weakly physio-adsorbed layer that was highly prone to detach from the biosensor surface during vigorous washing steps. However, in a later study where chitosan was used to entrap multiwall-carbon nanotube and graphene oxide nanoribbon mixture with the myoglobin (Mb) to generate a new species of biosensor targeting reactive oxygen species such as nitrites (NO^−^) and hydrogen peroxide (H_2_O_2_) the sensor construct demonstrate much better stability [13]. Thus, we hypothesized to resolve this challenge using a well-described covalent coupling technique to anchor chitosan (CH)-doped GONRs on the GCE surfaces. CH is a biopolymer commonly found in the shell of aquatic crustaceans. It is cost-effective, and highly bio-compatible. Furthermore, it contains excellent binding ability to physio-adsorb on GONRs creating a nanocomposite (GONRs-CH). Lastly, CH is rich in -NH_2_ moieties which were utilized for covalent attachment reactions. GCE was acid-activated to produce -COOH groups across the entire electrode surface; this allowed the common reaction of 1-ethyl-3-(3-dimethylaminopropyl) carbodiimide hydrochloride (EDC) and N-hydroxy succinimide (NHS) to produce a semi-stable amine-reactive -NHS ester cross-linker and follow react with the -NH_2_ groups within the CH matrix with GONR (GONRs-CH) to form amide bonds and immobilize on the surface of activated GCE. Since the GONRs were adsorbed onto CH polymer, CH bridged GONRs and GCE surface together via the -NH_2_ moiety. To the best of our knowledge, this approach using facile amine coupling technique to attach GONRs-CH onto GCE resulting a GCE/GONRs-CH assembly has yet been explored. Hence, this study presented a novel approach at attaching modifier composite to the electrode surface as a new approach to generate more stable electrochemical biosensors. Firstly, the obtained GONRs were characterized by field-emission scanning electron microscopy (FESEM) and high-resolution transmission electron microscopy (HRTEM). The stepwise modification of GCE are characterized by FTIR. Our results showed that GCE/GONRs-CH enabled the simultaneous and individual detection of all four DNA bases with a good limit of detection as well as maintaining an acceptable dynamic range. In addition, further experiments demonstrated that the sensor was stable and reproducible when challenged with real samples. 

## 2. Experimental

### 2.1. Materials and Methods

Chitosan (CH, 75% deacetylated), 1-ethyl-3-(3-dimethylaminopropyl) carbodiimide hydrochloride (EDC, 98%), N-hydroxysuccinimide (NHS, 98%), adenine (A, ≥99%), cytosine (C, ≥99%), thymine (T, ≥99%), guanine (G, 98%), potassium hexacyanoferrate(III) (K_3_[Fe(CN)_6_]), potassium permanganate (KMnO_4_, ≥99%), hydrogen peroxide solution (H_2_O_2_, 30 wt%) sulfuric acid (H_2_SO_4_, 95.0%–98.0%) and multi-walled carbon nanotubes (MWCNTs with OD of 20–30 nm with a wall thickness of 1–2 nm and length ranging from 0.5 to 2 μm and purity of >95%) were obtained from Sigma-Aldrich Company (Oakville, ON, Canada) and all chemicals were used as received. Phosphate electrolyte and buffer solution (PBS) at pH in the ranges 2.0 to 8.0 were prepared using H_3_PO_4_ (0.2 M) and the pH was adjusted with NaOH (1.0 M). All solutions were prepared in Milli-Q^®^ water with a resistivity of 18.2 MΩ.cm and a total organic carbon (TOC) < 5 ppb from Cascada LS water purification system (Pall Co., Mississauga, ON, Canada).

### 2.2. Instrumentation 

High-resolution transmission electron microscopy (HRTEM) and field-emission scanning electron microscopy (FESEM) imaging studies were performed on a Hitachi Field Emission Transmission Electron Microscope HF-3300 (Hitachi, Tokyo, Japan). Fourier-transform infrared spectroscopy (FTIR) analysis was performed using a Bruker Alpha ii with attached reflection model (Bruker, Massachusetts, United States). An aliquot (10 µL) of samples were drop-coated onto a carbon-coated copper grid (Electron Microscopy Science, Pennsylvania, USA). All electrochemical measurements were completed at room temperature using an Autolab PGSTAT 128N (EcoChemie, Utrecht, The Netherlands) potentiostat/galvanostat controlled by NOVA™ 2.1 software in connection with a conventional three-electrode cell including the nanocomposite-modified GCE as the working electrode and a platinum wire as the counter electrode with a saturated silver/silver chloride electrode (Ag/AgCl) as the reference electrode. All electrochemical impedance spectroscopy (EIS) measurements are performed in 10 mM [Fe(CN)_6_]^3−/4−^ with 0.10 M KCl over the frequency range from 0.1 Hz to 100 kHz with a 10 mV potential amplitude. Different pulse voltammetry (DPV) was performed from ™0.3 to 1.6 V at a step potential of 5 mV with a modulation amplitude of 0.025 V, a modulation time of 0.05 s and an interval time of 0.5 s.

### 2.3. Synthesis of Graphene Oxide Nanoribbons (GONRs) 

The oxidative unzipping of MWCNTs was achieved by following a modified version of the Tour method [14]. Briefly, an aliquot (50 mg) of MWCNTs was stirred in 50 mL concentrated H_2_SO_4_ for 12 h at room temperature. The mixture was kept mixing for another 1 h after adding 250 mg KMnO_4_ as an oxidant. Then, the oxidized mixture was heated at 55 °C in an oil bath for 30 min followed by raising the reaction temperature to 70 °C to stabilize the reactants. Afterwards, the reaction chamber was allowed to cool down to room temperature. The solution at room temperature was poured into 150 mL of water (in an ice bath at 0 °C) with 2 mL of H_2_O_2_ (37%) and centrifuged at 13,000 rpm for 15 min. The solid pellet was collected and then stirred in 50 mL water for 30 min and ultrasonicated for 15 min. An aliquot (6 mL) of H_2_O_2_ was added to the mixture and centrifuged again. The residues were stirred in 50 mL ethanol for 30 min and ultrasonicated for 15 min. An aliquot (50 mL) of diethyl ether was added to the mixture and centrifuged again. The products, GONRs, were washed in 25 mL of ether twice and dried at room temperature. 

### 2.4. Preparation of Nanocomposite-Modified GCE

**Scheme 1:**
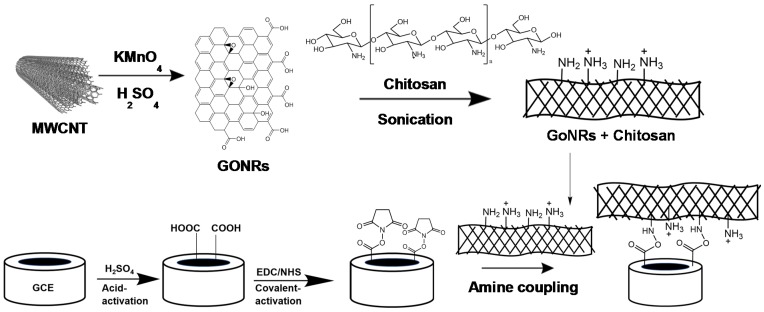
Schematic illustration of the preparation of graphene oxide nanoribbons (GONRs) using acid activation and oxidation with KMnO_4_ followed by ultra-sonication in chitosan (CH) solution. The nanocomposite was attached with amine coupling reaction between the primary amine groups of CH and active N-hydroxy succinimide (NHS) esters anchored on the activated glassy carbon electrode (GCE) surface.

GCEs were first polished with alumina powder at different particle sizes of 1.0 µm, 0.3 µm and 0.05 µm for 15 min for each size. The polished GCEs were then sonicated in water to remove the excess alumina particles. The acid-activation then proceeded in 0.5 M H_2_SO_4_ in the potential range from −1.0 to 1.5 V at a scan rate of 100 mV/s for 30 cycles. After gently rinsing with water, the preparation of acid-activated GCEs was completed. The activated GCEs were modified with 150 mM 1-ethyl-3-(3-dimethylaminopropyl) carbodiimide hydrochloride (EDC) dissolved in water for 1 h, then dipped in 300 mM N-hydroxy succinimide (NHS) for 2 h and dried with flowing stream of N_2_ (purity 99.999%). The stock solution of CH (0.05% (w/v)) was prepared by mixing 500 mg CH powder in 1 L water and 10 mL acetic acid. The stock solution of CH was stirred for 1 h using a magnetic stirrer. The nanocomposite was prepared by mixing a desired amount of GONRs (2 mg) in 2 mL of CH followed by ultra-sonication for 10 min. The covalently-activated GCE surfaces were then modified by drop-casting an aliquot (20 μL) of the freshly prepared nanocomposite solution. The modification steps are illustrated in Scheme 1. The nanocomposite-modified electrodes were allowed to dry overnight at room temperature and then gently rinsed with water. 

### 2.5. Preparation of DNA Base Stock Solutions

The stock solutions of 0.01 M G, A, T and C nucleobases were prepared in 5 mL water with 100, 20 and 60 µL of 9.0 M NaOH solution for dissolving of G, A and T, respectively.

## 3. Results and Discussion

### 3.1. Characterization of GONRs

**Figure 1:**
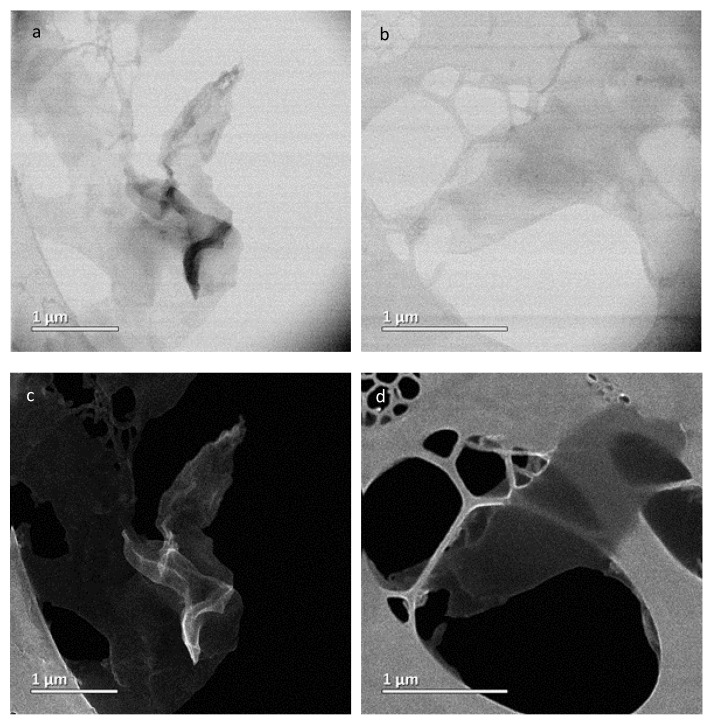
High-resolution transmission electron microscopy (HRTEM) (**a**,**b**) and field-emission scanning electron microscopy (FESEM) (**c**,**d**) images of GONRs with scale bar of 1 µm.

To demonstrate the successful synthesis of GONRs, HRTEM and FESEM imaging studies were performed. Shown in Figure 1a,b is various images of the GONRs. As demonstrated, the unraveling process of the MWCNTs was observed, where sheet like structure were present. As shown in Figure 1c,d, the FESEM of graphene nanoribbons continued to appear as sheet-like structures compared to the tubular structures observed in MWCNTs (shown in Figure S6, in the Supplementary Materials) which was similar to what was previously observed [15]. Thus, demonstrating the synthesis of GONRs was successful. 

### 3.2. Characterization of GCE and Stepwise GCE Modification

**Figure 2:**
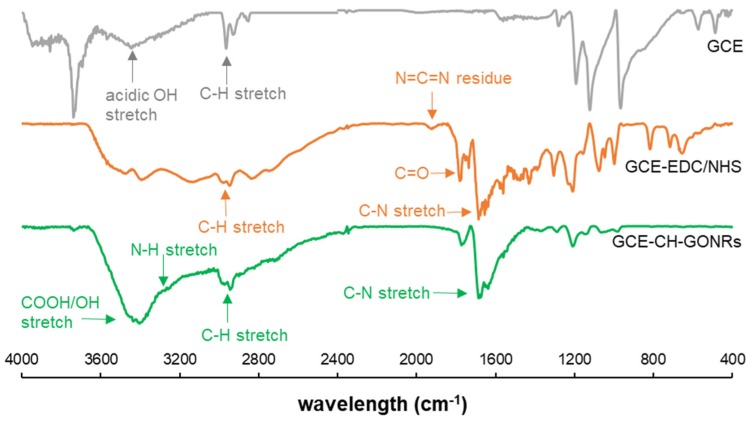
The FTIR spectra of GCE (grey), GCE post 1-ethyl-3-(3-dimethylaminopropyl) carbodiimide hydrochloride (EDC)/NHS activation (orange) and GCE-EDC/NHS-CH-GONR (green).

To show the corresponding modification steps were successful, FTIR analysis of the electrode surface was performed. As shown in Figure 2, the acid activated glassy carbon electrode presented the acidic OH stretch and C-H stretch at 3441 and 2963 cm^−1^, respectively. A free OH stretch was also observed at 3733 cm^−1^ indicating the electrode surface were presented with the expected -COOH group. After amine coupling activation using EDC and NHS, a C=O stretch was observed around 1777 cm^−1^ and a small peak around 1918 cm^−1^ suggesting the N=C=N functional group from EDC, which was previously reported [16]. After attaching the chitosan and GONRs (CH-GONRs) composite, the N=C=N peak disappeared, and with the addition of CH-GONRs composite, a broad peak correlated to -COOH was found at 3400 cm^−1^ corresponding to the carboxyl and hydroxyl functional groups found on GONRs. A peak was found at 1774, which suggests the presence of amide moieties similar to the previous report [17]. Lastly, a small N-H stretch shoulder peak appeared around 3250 cm^−1^, which corresponds to the presence of N-H from the chitosan. Conclusively, from the FTIR results, the modification process was successful.

### 3.3. Electrochemical Characterization of GCE/GONRs-CH

**Figure 3:**
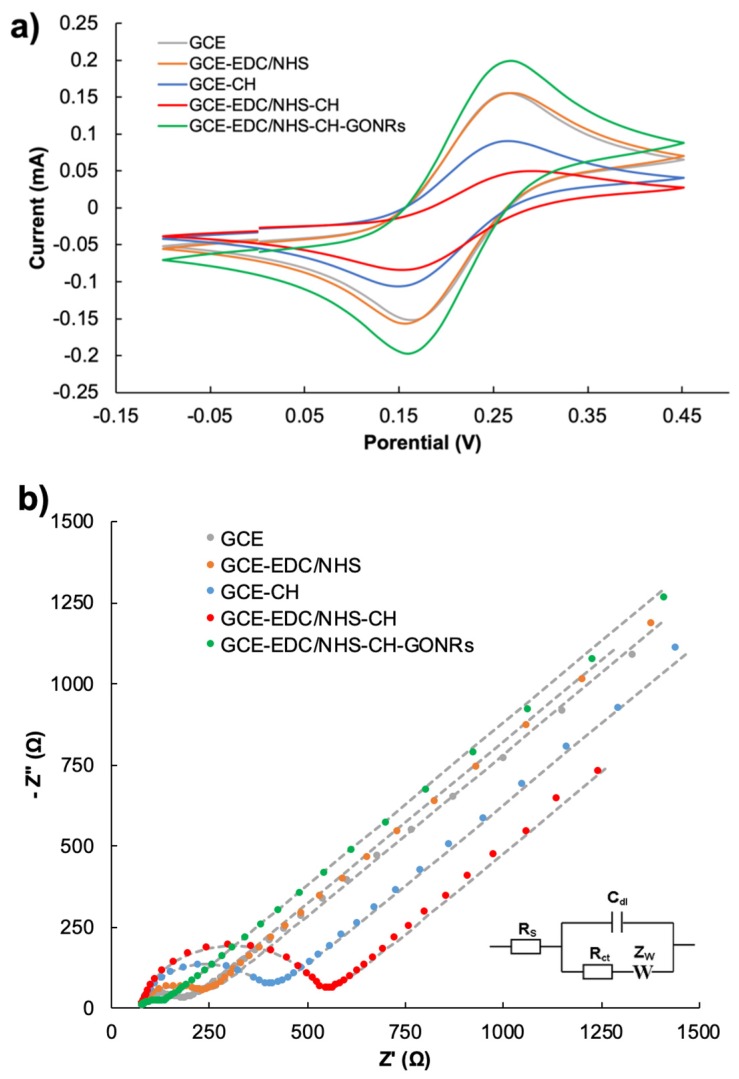
Cyclic voltammograms (**a**) and the Nyquist plots (**b**) of GCEs after various modifications. GCE (grey), GCE/CH (orange) and GCE/GONRs-CH (green). EIS measurements were performed in 10 mM [Fe(CN)_6_]^3−/4−^ with 0.10 M KCl over the frequency range from 0.1 Hz to 100 kHz with a potential of 0.28 V vs. Ag/AgCl on a 10 mV potential amplitude. R_ct_ values were obtained by the fitting of the Nyquist plot with the modified Randle equivalent circuit (shown in b, inset).

To monitor the modification process as well as the electrochemical activity of GCE/GONRs-CH, CV and EIS were performed. In Figure 3, the bare GCE showed an anodic and cathodic peak for [Fe (CN)_6_]^3−/4−^ at 0.27 V and 0.15 V (vs. Ag/AgCl), respectively (Figure 3a). CV of the modified GCE with EDC/NHS was similar to GCE but when the GCE modified with CH and EDC-NHS-CH, the anodic potential increased with a decrease in the reduction potential for both modified electrodes but the increasing anodic potential for GCE/EDC-NHS-CH was more than that of GCE-CH. This result supported the successful attachment of CH on GCE surface. The GCE/GONRs-CH displayed an obvious increase in anodic and cathodic currents while confirming the incorporation of GONRs in CH. EIS (Figure 3b) was also performed after the CV scans, and the results are displayed as Nyquist plots fitted with a modified Randles equivalent circuit. Firstly, for bare GCE and the modified GCE with EDC/NHS amine coupling agents, the corresponding charge-transfer resistance (R_ct_) values showed no observable differences. After the incorporation of CH on the surface, the R_ct_ increased. This was expected as CH was not modified with any redox-active components. This was reflected (as shown in Figure 3b) in the corresponding Nyquist plots, where an increase of R_ct_ from 473.47 Ω to 180.14 Ω was observed for GCE-CH to GCE, respectively. For the GCE where the surface was activated with EDC/NHS and CH was introduced, the R_ct_ further increased. This suggested that the amine coupling reaction allowed for more CH to attach to the electrode surface. Lastly, the modified GCE/GONRs-CH displayed a decrease in R_ct_ with an increase in the current density in CV study. This was attributed to the electrocatalytic property of GONRs to facilitate electron transfer.

### 3.4. Simultaneous Detection of Guanine, Adenine, Thymine and Cytosine 

**Figure 4:**
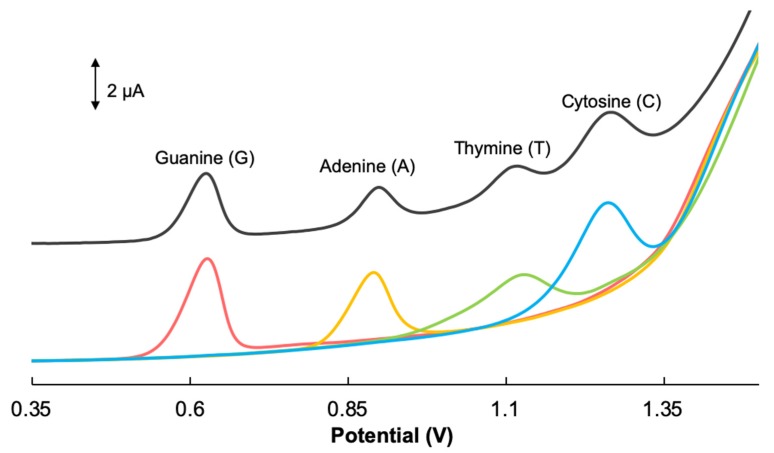
Differential pulse voltammograms of GCE/GONRs-CH (60 µM, red line), G (12 µM, orange line), A (12 µM, yellow line), T (170 µM, green line) and C (170 µM, blue line) individually and in the mixture (black line) in 0.1 M PBS (pH 7.0).

It was important to examine the performance of the GCE/GONRs-CH in both separate and mixed solutions of nucleobases. As shown in Figure 4, DPV peaks were found at 0.63, 0.89, 1.13 and at 1.27 V which were corresponding to the oxidation potentials of G (orange line), A (yellow line), T (green line) and C (blue line), respectively. When all four nucleobases were mixed, as shown on the differential pulse voltammogram, the electrochemical oxidation signals correlating to all four nucleobases were detected (Figure 4-black line) in a single scan. Four nucleobases were detected with excellent peak separation. This demonstrated that GCE/GONRs-CH provided a promising sensor surface for detecting four nucleobases simultaneously as well as individually.

**Figure 5:**
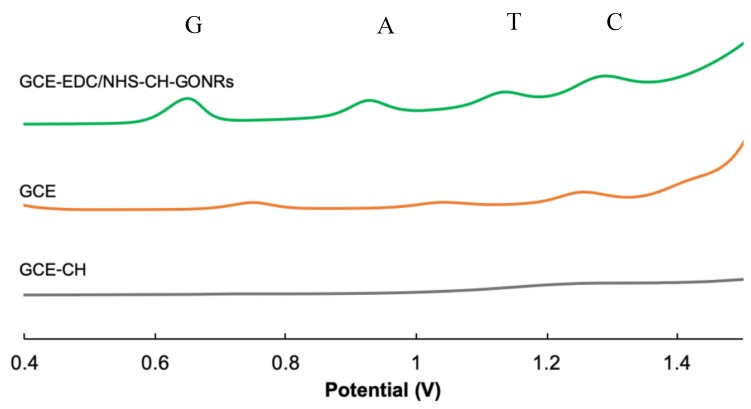
Different pulse voltammetry (DPV) of the GCEs after various modification steps in the presence of G, A, T and C at GCE-CH (grey line), GCE (orange line) and GCE/GONRs-CH (green line) in 0.1 M PBS (pH 7.0) containing 0.001 M of nucleobase solutions.

As shown in Figure 5, when detecting four nucleobases using a bare GCE, low and board peaks were recorded for the nucleobases. With the incorporation of the CH at GCE/CH, the peaks for the nucleobases completely became non-observable. This was expected as CH did not have redox-active components incorporated to facilitate the electron transfer. Finally, at GCE/GONRs-CH, the peaks for the four nucleobases became observable. Henceforth, the modification of GCE with GONRs-CH nanocomposite was shown to greatly enhance the electrochemical performance.

### 3.5. pH Effect

**Scheme 2:**
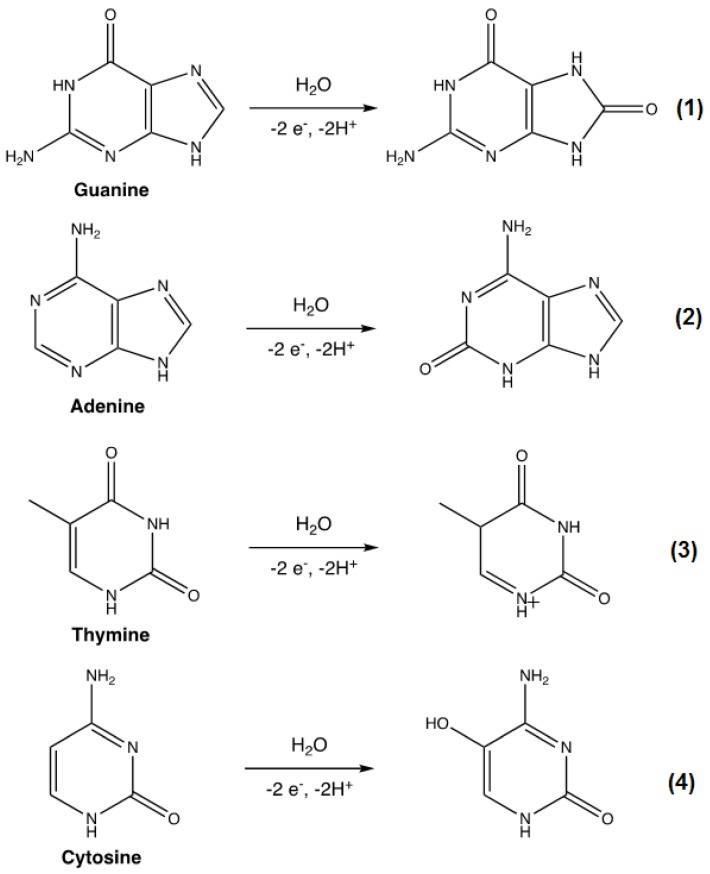
The electrochemical oxidation of nucleobases.

The effect of the pH environment on the electrochemical detection performance of GCE/GONRs-CH in the presence of four nucleobases was analyzed. As shown in Figure S1a, the peak potentials were shifted towards lower potentials as pH increased. In addition, the peak currents for nucleobases were increased with increasing the pH from 2.0 to 7.0 and then decreased when pH reached to 8.0. Based on the DPV studies, pH 7.0 displayed the highest peak currents with the best peak separation. Thus, it was determined to be the optimal pH environment. In addition, to further understand the mechanism of the electron-proton exchange processes, the potential shift was plotted against the pH and the results are shown in Figure S1b. By fitting the plotted values, a slope was interpreted for each nucleobase and they were found to be 0.0472, 0.0488, 0.0473 and 0.0473 V/pH for G, A, T and C, respectively. This complies with the Nernstian value of 0.059 V/pH. Therefore, this suggested the electrochemical oxidation of nucleobases followed an equal ratio of proton to electron exchange mechanism as described by previous reports in the following Scheme 2 [18,19,20]. 

### 3.6. Calibration Curves

**Figure 6:**
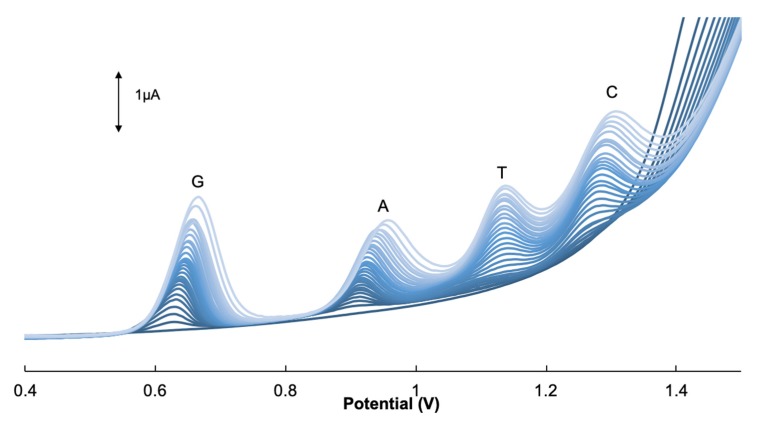
Differential pulse voltammograms of GCE/GONRs-CH in the presence of mixed solutions with G (0.05–256 µM), A (0.05–172 µM), T (5.0–855 µM) and C (2.5–342 µM) in 0.1 M PBS (pH 7.0).

To correlate the signal response to the concentration of the nucleobase, a calibration curve was constructed. As shown in Figure 6, the DPVs depicting the current density of the four DNA bases as it is simultaneously added into the solution. The GCE/GONRs-CH sensor demonstrated a linear response to the analyte added. The peak currents were extrapolated and plotted against the concentration and the results are shown in Figure S2 and summarized in Table 1. Two segment linear regression curves were found with the exception of C with one segment. The calibration curves were linear up to 256, 172, 855 and 342 µM with a limit of detection (LOD= 3S_bk_/m where S_bk_ and m are standard division of blank solution for 10 determinations and slope of calibration curves, respectively) 0.0018, 0.0231, 1.3304 and 0.6406 µM for G, A, T and C, respectively. The figures of merit of the nanocomposite-modified sensor in comparison with the reported ones in literature for simultaneous determination of G, A, T and C are shown in Table 2. Based on Table 2, the linear ranges and LOD of GCE/GONRs-CH were more improved as well as comparable with those reported ones for the electrochemical detection of all four nucleobases.

The cross-interference studies for the simultaneous determination of G, A, T and C were also performed and the results are shown in Figure S3. In this study, the concentration of three nucleobases were held constant, while the concentration of one of the nucleobases was increased in the mixture. Based on Figure S3, the relative percent signal deviations were calculated as 3.29, 3.07, 2.59 and 2.86% for G, A, T and C, respectively.

### 3.7. Reproducibility and Stability

To demonstrate that GCE/GONRs-CH provided stable and reproducible results, multiple measurements in the presence of G (30 µM), A (30 µM), T (300 µM) and C (170 µM) were performed. As shown in Figure S4, the current peaks for A, T, G and C demonstrated a relative standard deviation of 4.7%, 2.7%, 4.6% and 2.24%, respectively (n = 10). The stability of GCE/GONRs-CH was determined by storing the nanocomposite-modified electrodes at room temperature over a period of 3 weeks and the results are shown in Figure S5. Consecutive measurements were taken at week-3 and compared to the ones recorded initially on the first day of week-1. The I_pa_ values were calculated to be retained at 98.5, 93.1, 91.9 and 96.9% compared to the initial responses recorded for the electrochemical oxidation signal of G, A, T and C, respectively. 

### 3.8. Real Samples 

To determine the real sample applications of GCE/GONRs-CH, it was subjected to detect the four nucleobases G, A, T and C in real samples. To understand the effect that the sugar backbone might have towards the electrochemical detection, the first sample was prepared to detect the nucleobases in the mononucleotide form. Two types of real samples of nucleotides and dsDNA were used for simultaneous determination of G, A, T and C. For the first sample, the DNA mononucleotides were pretreated to release the DNA bases from the glycoside bonds as described by previous literature [28]. Briefly, 1 mg/mL of DNA mononucleotides was dissolved in 1 mM of HCl and then the solutions were boiled in a water bath for 2 h. The pH was then adjusted using 1 M NaOH to pH 7.0 and stored in −20 °C before use. For dsDNA samples, a digestive process was used to break the double-stranded helices and release the DNA bases as described previously [29]. In this method, 1 mg of calf thymus dsDNA sample was dissolved in 1 mL of 1 M HCl solution and then, the solution was heated to boil under stirring for 2 h. The solution was cooled down to room temperature, and further neutralized to pH 7.0 using 5 M NaOH and stored in −20 °C before use. The results for simultaneous determinations of these two pretreated real samples are summarized in Table 3. In this table, the “Detected” is the analyte concentration before standard solution addition. “Spiked” indicates the concentration of analyte added into the solution, and finally “Determined” is the analyte concentration that was found after correlating the signal obtained from the sensor to the presented linear regression curve (*n* = 3). The results for the first real sample demonstrated a recovery percentage between 95.4 to 97.9%. This demonstrated that the sensor platform was able to detect the nucleobases in mononucleotide form. In addition, a recovery percentage was observed from 91.14% to 104.7% for the second real sample. Before the standard addition, it was expected the matching pair G and C or A and T should have a similar concentration. However, it was found that the detected concentrations fluctuated between the two sets of matching nucleotides. This was attributed to the pre-treatment process, where the calf thymus DNA was subjected to acid treatment to denature the nucleotides and release them from the double-helical form. Thus, it is likely that during the denaturation processes resulted in loss of analytes, henceforth an unequal concentration was observed. Nevertheless, it was noteworthy that these preliminary results demonstrated GCE/GONRs-CH as a promising biosensor to detect all four DNA bases in real samples after pre-treatment.

## 4. Conclusions

In this study, we have modified a GCE with a novel nanocomposite of CH and GONRs. Using common EDC/NHS amine coupling activation of GCE surfaces, the nanocomposite was successfully attached on the surfaces as displayed by the CV and EIS studies. The modification of GCE with the nanocomposite displayed a significant increase in current signal intensity compared to bare GCE and GCE/CH. Finally, GCE/GONRs-CH displayed a stable and reproducible signal intensity over several weeks of storage and demonstrated acceptable recovery values with the detection of simultaneous detection of the four nucleobases in real samples after pretreatment procedures of DNA. With further modification and improvement, this nanocomposite-modified GCE can be developed into the next generation of new biosensors for the detection of DNA damage and mutations. 

## Figures and Tables

**Table 1:** Summary of the calibration curve and the R^2^ values of the linear fit, the limit of detection and the dynamic range for the electrochemical detection of four nucleobases using GCE/GONRs-CH.

Analyte	I	II	LOD (µM)	Dynamic Range (µM)
**G**	ΔI_p1_ = 0.3651x + 0.066 R² = 0.9919	ΔI_p2_ = 0.0621x + 1.493 R² = 0.9901	0.002	0.05–256.0
**A**	ΔI_p1_ = 0.1963x + 0.144 R² = 0.9911	ΔI_p2_ = 0.0606x + 0.881 R² = 0.9916	0.023	0.05–172.0
**T**	ΔI_p1_ = 0.008x + 0.4086 R² = 0.9944	ΔI_p2_ = 0.0008x + 2.947 R² = 0.9923	1.330	5.0–855.0
**C**	ΔI_p_ = 0.0186x + 0.4199 R² = 0.9909		0.641	2.5–342.0

**Table 2:** Analytical performance of GCE/GONRs-CH in comparison with those reported in the literature.

Analyte	Modified Working Electrode	LOD (µM)	Dynamic Range (µM)	Ref.
**G**	GCE	0.06	0.2–10	[21]
	CILE ^a^	0.00787	0.3–50	[22]
	Pretreated GCE ^b^	0.03	0.08–2.7	[23]
	TiO_2_-graphene/GCE	0.15	0.5–200	[24]
	GCE/GONRs-CH	0.0018	0.013–256	This study
**A**	GCE	0.07	0.2–10	[21]
	CILE	0.25	1.5–70	[22]
	Pretreated GCE	0.03	0.08–2.7	[23]
	TiO_2_-graphene/GCE	0.10	0.5–200	[24]
	PTH/NPAu/MWNTs ^c^	0.008	0.05–5	[25]
	GCE/GONRs-CH	0.023	0.11–172	This study
**T**	GCE	0.89	1–20	[21]
	PANI/MnO_2_^d^	1.3	10–100	[9]
	α-CD/CNT/CE ^e^	5	25–1800	[26]
	PEDOT/GR/GCE ^f^	0.33	2.5–650	[7]
	GCE/GONRs-CH	1.330	6.0–855	This study
**C**	GCE	1.76	1–20	[21]
	PANI/MnO_2_	1.3	10–100	[9]
	CPE ^g^	1.9	10–1000	[27]
	PEDOT/GR/GCE	0.33	1–1000	[7]
	GCE/GONRs-CH	0.640	3.5–342	This study

^a^ Carbon Ionic Liquid electrode. ^b^ Glassy carbon electrode activated with phosphate buffer (pH 5.0). ^c^ Polythionine-gold nanoparticles and multi-wall carbon nanotubes. ^d^ Polyaniline and manganese oxide. ^e^ α-Cyclodextrin incorporated carbon nanotube coated electrode. ^f^ Poly(3,4-ethylenedioxythiophene)-graphene oxide composite film glassy carbon electrode. ^g^ Carbon paste electrode.

**Table 3:** Application of GCE/GONRs-CH for simultaneous determination of G, A, T and C in the two real samples of single nucleotides and dsDNA (*n* = 4, for the reported RSD values).

	**Analyte**	**Detected (µM)**	**Spiked (µM)**	**Determined (µM)**	**Recovery (%)**
**Single Nucleotides**	A	25.9 ± 3.89	25	23.85 ± 0.30	95.41 ± 1.2
	T	24.4 ± 4.92	50	48.91 ± 1.03	97.90 ± 2.06
	G	24.9 ± 2.87	25	24.08 ± 0.22	96.34 ± 0.88
	C	22.5 ± 1.10	15	14.44 ± 0.44	96.28 ± 2.93
**dsDNA**	A	13.04 ± 1.47	25	23.80 ± 0.13	95.26 ± 0.52
	T	11.38 ± 2.28	40	39.8 ± 1.37	99.50 ± 3.4
	G	19.23 ± 1.10	25	22.8 ± 0.39	91.14 ± 1.7
	C	23.75 ± 2.79	20	20.9 ± 2.75	104.7 ± 13.8

## Supplementary Materials

Differential pulse voltammograms voltammograms of pH dependence, calibration plots, differential pulse voltammograms of interference studies, reproducibility and stability studies are available online at https://www.mdpi.com/2079-6374/10/4/30/s1, Figure S1. (a) Differential pulse voltammograms of GCE/GONRs-CH surfaces in 0.1 M PBS with the presence of G (70 μM), A (70 uM), T (290 μM) and C (290 μM). (b) The dependence of peak potentials of four nucleobases on pH. Figure S2. The calibration plots of G (a, 0.05–256 µM), A (b, 0.05-172 µM), T (c, 5–855 µM) and C (d, 2.5–342 µM) with the concentration plotted against the anodic peak current. Figure S3. Interference studies of four analytes. Figure S4. DPVs of 10 consecutive runs on the GCE/GONRs-CH in the presence of 10 µM G, 10 µM A, 80 µM T and 80 µM C in 0.1 M PBS (pH 7.0). Figure S5. DPVs of GCE/GONRs-CH in the presence of 50 µM G, 50 µM A, 100 µM T and 100 µM C at week 1 (blue) and week 3 (orange) in 0.1 M PBS (pH 7.0). Figure S6. HRTEM image of MWCNTs with the scale bar of 20 nm.
