# Supplementary material for: Graphene Oxide Nanoribbons in Chitosan for Simultaneous Electrochemical Detection of Guanine, Adenine, Thymine and Cytosine
Source: Biosensors (Basel). 2020 Mar 27;10(4):30. doi: 10.3390/bios10040030 (PMC7236021; doi:10.3390/bios10040030)
Supplement: Supplementary file 1 [file biosensors-10-00030-s001.pdf]

Supplementary Information

for:

Graphene oxide nanoribbons in chitosan for simultaneous electrochemical detection of guanine, adenine, thymine and cytosine

By;

Jiayun Zhou[†], Shaopei Li[†], Meissam Noroozifar and Kagan Kerman[*]

Department of Physical and Environmental Sciences, University of Toronto Scarborough,
1265 Military Trail, Toronto, ON, M1C 1A4, Canada

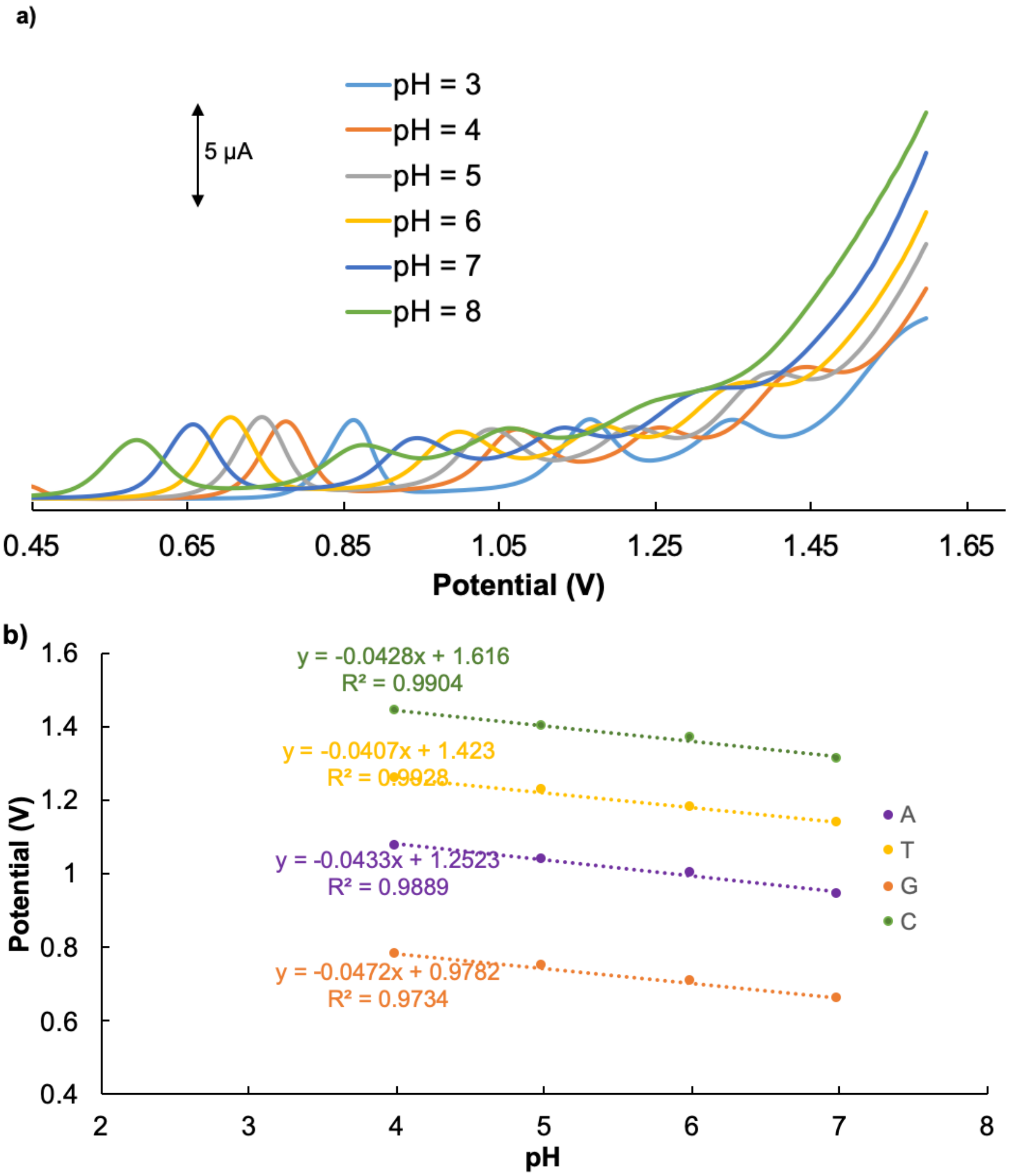

Figure S1. (**a**) Differential pulse voltammograms of GCE/GONRs-CH surfaces in 0.1 M PBS at pH 3.0 (purple line), 4.0 (red line), 5.0 (black line), 6.0 (yellow line), 7.0 (blue line) and 8.0 (green line) with the presence of G (70 μM), A (70 uM), T (290 μM) and C (290 μM). (**b**) The dependence of peak potentials of four nucleobases on pH.

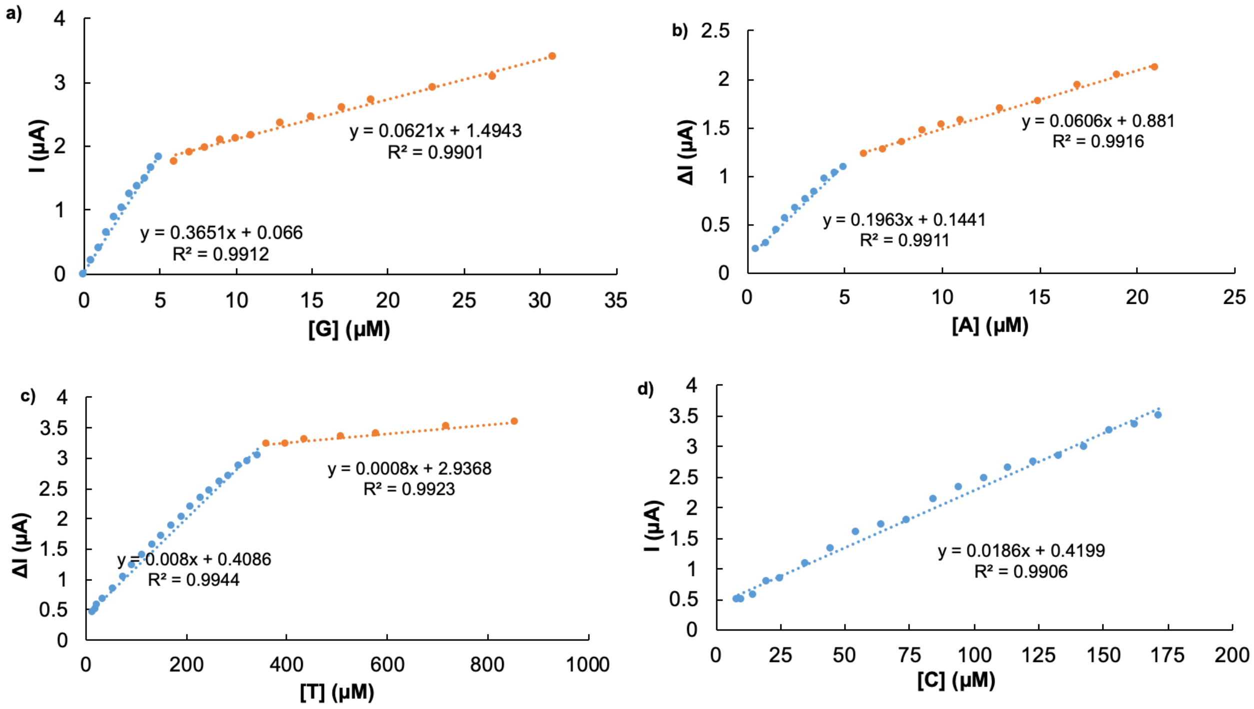

Figure S2. The calibration plots of G (**a**, 0.05-256 μM), A (**b**, 0.05-172 μM), T (**c**, 5-855 μM) and C (**d**, 2.5-342 μM) with the concentration plotted against the anodic peak current.

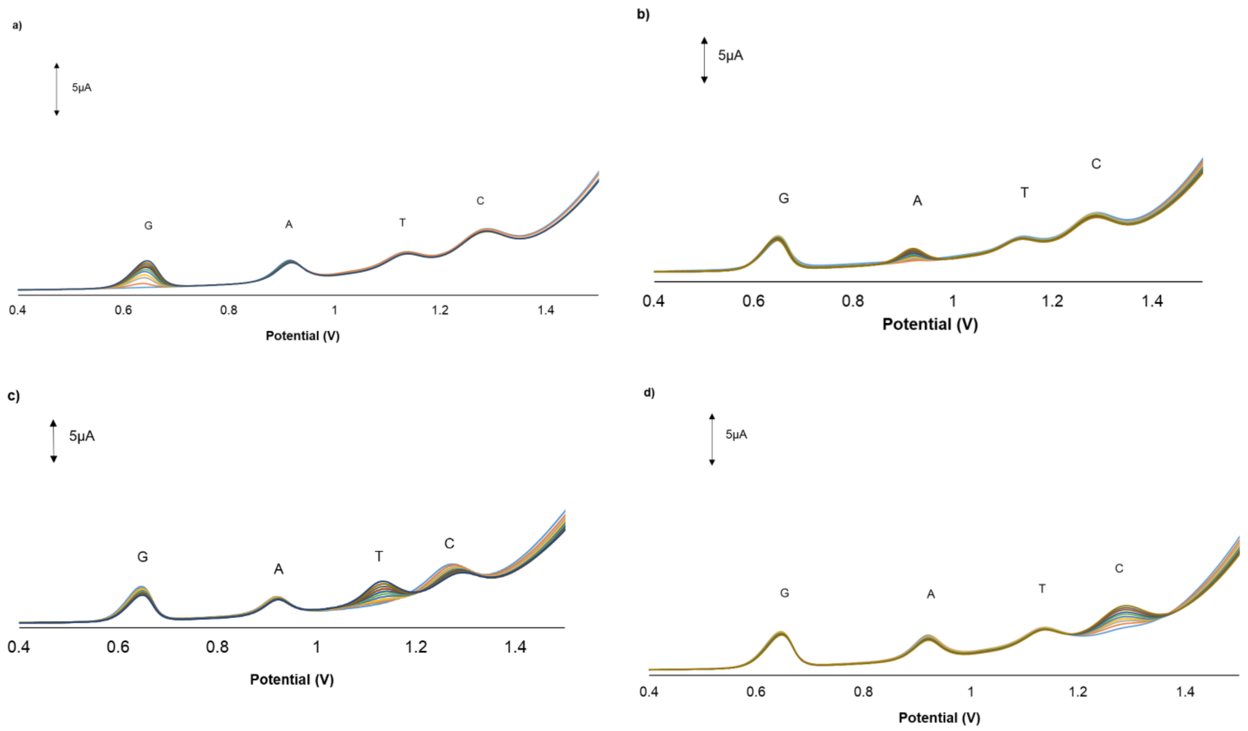

Figure S3. Interference studies of four analytes (**a**) G (0.5-1 µM) A (20 µM), T (80 µM.) and C (75 µM); (**b**) A (2-20 µM) G (20 µM), T (80 µM) and C (75 µM); (**c**) T (20-500 µM) G (20 µM), A (20 µM) and C (75 µM); (**d**) C (20-300 µM), G (20 µM), A (20 µM) and T (80 µM), using GCE/GONRs-CH surfaces in 0.1 M PBS (pH 7.0).

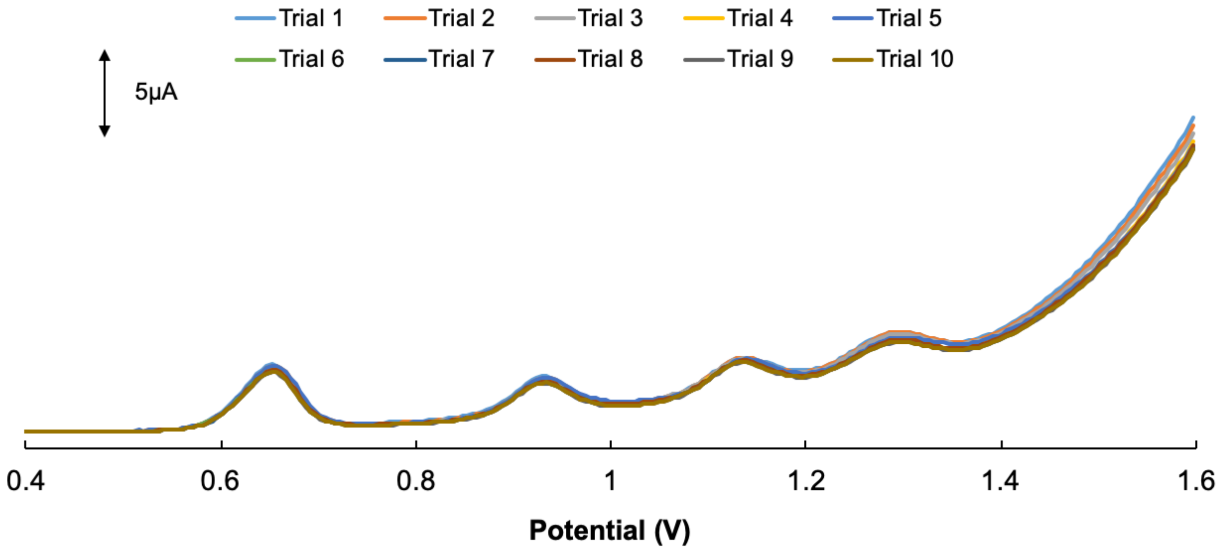

Figure S4. DPVs of 10 consecutive runs on the GCE/GONRs-CH in the presence of 10 μM G, 10 μM A, 80 μM T and 80 μM C in 0.1 M PBS (pH 7.0).

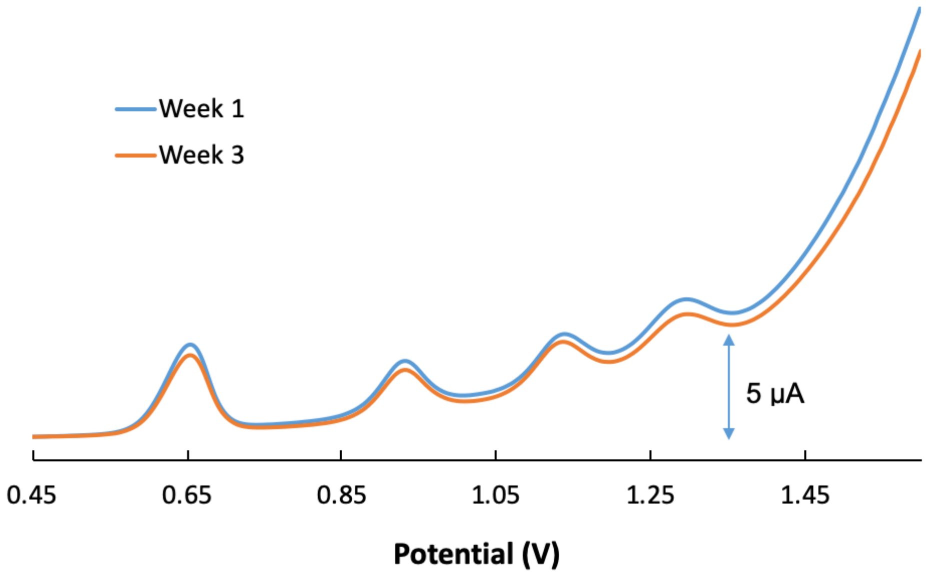

Figure S5. DPVs of GCE/GONRs-CH in the presence of 50 µM G, 50 µM A, 100 µM T and 100 µM C at week 1 (blue) and week 3 (orange) in 0.1 M PBS (pH 7.0).

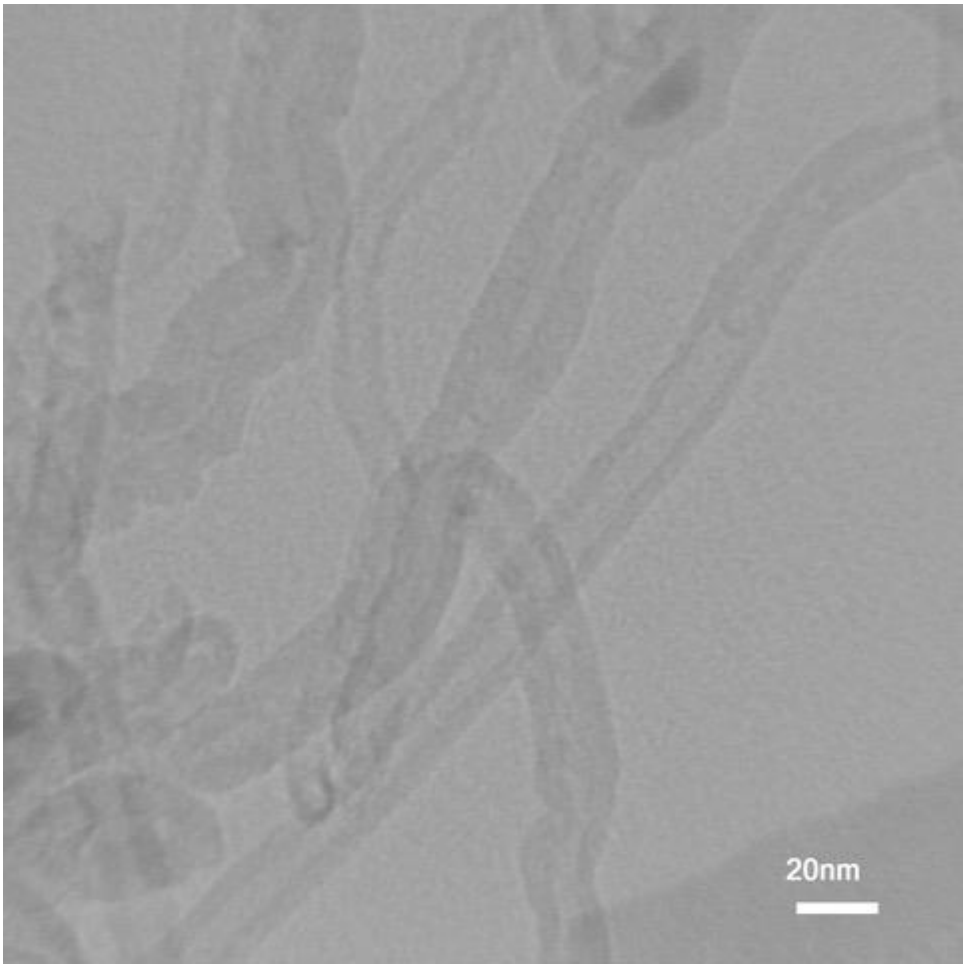

Figure S6. HRTEM image of MWCNTs with the scale bar of 20 nm.
